# Gene model for the ortholog of *DENR* in *Drosophila yakuba*

**DOI:** 10.17912/micropub.biology.001017

**Published:** 2024-11-12

**Authors:** Leon F. Laskowski, Inayah Burton, Timothy J. Stanek, Geoffrey D. Findlay, Scott Tanner, Jack A. Vincent, Solomon Tin Chi Chak, Christopher E. Ellison, Chinmay P. Rele

**Affiliations:** 1 University of Alabama, Tuscaloosa, Alabama, USA; 2 SUNY Old Westbury, Old Westbury, NY USA; 3 Rutgers University, New Brunswick, NJ USA; 4 College of the Holy Cross, Worcester, MA USA; 5 University of South Carolina Upstate, Spartanburg, SC USA; 6 University of Washington - Tacoma, Tacoma, WA USA

## Abstract

Gene model for the ortholog of *Density regulated protein* ( *DENR* ) in the May 2011 (WUGSC dyak_caf1/DyakCAF1) Genome Assembly (GenBank Accession: GCA_000005975.1 ) of *Drosophila yakuba* . This ortholog was characterized as part of a developing dataset to study the evolution of the Insulin/insulin-like growth factor signaling pathway (IIS) across the genus *Drosophila* using the Genomics Education Partnership gene annotation protocol for Course-based Undergraduate Research Experiences.

**Figure 1.**
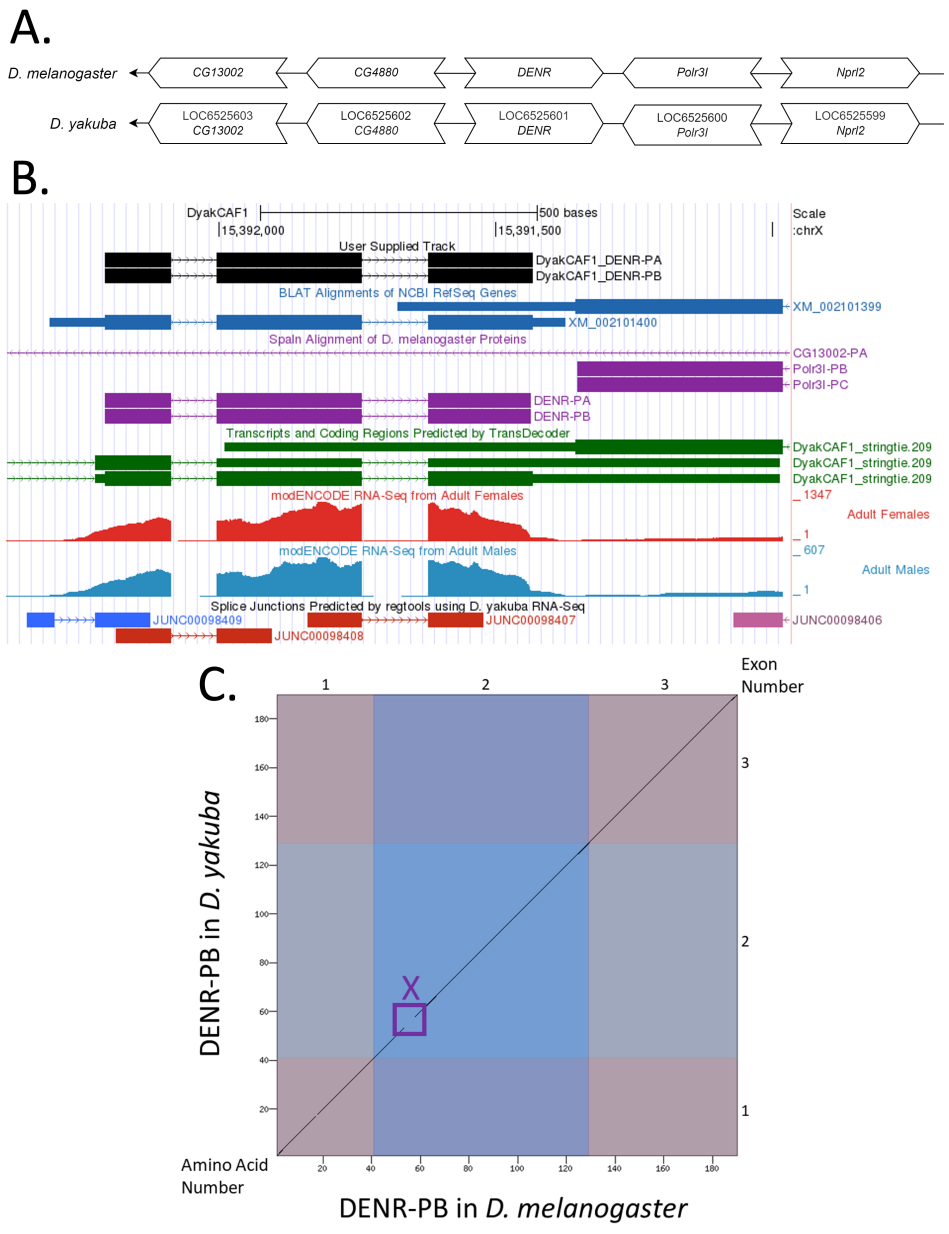
**
(A) Synteny comparison of the genomic neighborhoods for *DENR* in *Drosophila melanogaster* and *D. yakuba* .** Thin underlying arrows indicate the DNA strand within which the target gene– *DENR* –is located in *D. melanogaster* (top) and *D. yakuba* (bottom). The thin arrows pointing to the left indicate that *DENR* is on the negative (-) strand in *D. yakuba* and *D. melanogaster* . The wide gene arrows pointing in the same direction as *DENR* are on the same strand relative to the thin underlying arrows, while wide gene arrows pointing in the opposite direction of *DENR* are on the opposite strand relative to the thin underlying arrows. White gene arrows in *D. yakuba* indicate orthology to the corresponding gene in *D. melanogaster* . Gene symbols given in the *D. yakuba* gene arrows indicate the orthologous gene in *D. melanogaster* , while the locus identifiers are specific to *D. yakuba* . **(B) Gene Model in GEP UCSC Track Data Hub (Raney et al., 2014).** The coding-regions of *DENR* in *D. yakuba* are displayed in the User Supplied Track (black); CDSs are depicted by thick rectangles and introns by thin lines with arrows indicating the direction of transcription. Subsequent evidence tracks include BLAT Alignments of NCBI RefSeq Genes (dark blue, alignment of Ref-Seq genes for *D. yakuba* ), Spaln of *D. melanogaster* Proteins (purple, alignment of Ref-Seq proteins from *D. melanogaster* ), Transcripts and Coding Regions Predicted by TransDecoder (dark green), RNA-Seq from Adult Females and Adult Males (red and light blue, respectively; alignment of Illumina RNA-Seq reads from *D. yakuba* ), and Splice Junctions Predicted by regtools using *D. yakuba* RNA-Seq ( SRP006203 ). Splice junctions shown in blue (JUNC00098409) or red (JUNC00098408; JUNC00098407) have read-depths of 21, 1039 and 1780, respectively. **(C) Dot Plot of DENR-PB in *D. melanogaster* ( *x* -axis) vs. the orthologous peptide in *D. yakuba* ( *y* -axis).** Amino acid number is indicated along the left and bottom; CDS number is indicated along the top and right, CDSs are also highlighted with alternating colors. The purple box, X, indicates a lack of sequence similarity between amino acids.

## Description

**Table:** 

* This article reports a predicted gene model generated by undergraduate work using a structured gene model annotation protocol defined by the Genomics Education Partnership (GEP; thegep.org ) for Course-based Undergraduate Research Experience (CURE). The following information may be repeated in other articles submitted by participants using the same GEP CURE protocol for annotating Drosophila species orthologs of Drosophila melanogaster genes in the insulin signaling pathway. * "In this GEP CURE protocol students use web-based tools to manually annotate genes in non-model *Drosophila* species based on orthology to genes in the well-annotated model organism fruitfly *Drosophila melanogaster* . The GEP uses web-based tools to allow undergraduates to participate in course-based research by generating manual annotations of genes in non-model species (Rele et al., 2023) . Computational-based gene predictions in any organism are often improved by careful manual annotation and curation, allowing for more accurate analyses of gene and genome evolution (Mudge and Harrow 2016; Tello-Ruiz et al., 2019) . These models of orthologous genes across species, such as the one presented here, then provide a reliable basis for further evolutionary genomic analyses when made available to the scientific community.” (Myers et al., 2024) . “The particular gene ortholog described here was characterized as part of a developing dataset to study the evolution of the Insulin/insulin-like growth factor signaling pathway (IIS) across the genus *Drosophila* . The Insulin/insulin-like growth factor signaling pathway (IIS) is a highly conserved signaling pathway in animals and is central to mediating organismal responses to nutrients (Hietakangas and Cohen 2009; Grewal 2009) .” (Myers et al., 2024) . “ *D. yakuba* (NCBI:txid 7245) is part of the *melanogaster* species group within the subgenus *Sophophora* of the genus *Drosophila* (Sturtevant 1939; Bock and Wheeler 1972) . It was first described by Burla (1954). *D. yakuba* is wide-spread in sub-Saharan Africa and Madagascar (Lemeunier et al., 1986; https://www.taxodros.uzh.ch , accessed 1 Feb 2023; Markow and O'Grady 2006) where figs served as a primary host along with other rotting fruits (Lachaise and Tsacas 1983) .” (Koehler et al., 2024) .

We propose a gene model for the *D. yakuba* ortholog of the *D. melanogaster* *Density regulated protein* ( *DENR* ) gene. The genomic region of the ortholog corresponds to the uncharacterized protein LOC6525601 (RefSeq accession XP_002101436.1 ) in the Dyak_CAF1 Genome Assembly of *D. yakuba* (GenBank Accession: GCA_000005975.1 - Graveley et al., 2011). This model is based on RNA-Seq data from *D. yakuba* ( SRP006203 ) and *DENR* in *D. melanogaster* using FlyBase release FB2022_04 ( GCA_000001215.4 ; Larkin et al., 2021).

Density regulated protein was first discovered in a human teratocarcinoma cell line because its concentration in cells increased with cell density (Deyo et al., 1998) . Subsequent bioinformatic and biochemical analyses showed that the protein is conserved across eukaryotes and functions in non-canonical translation initiation (Fleischer et al., 2006; Skabkin et al., 2010) . *D. melanogaster* flies homozygous for a null, knockout allele of the gene encoding *Density regulated protein* , *DENR* (FBgn0030802), die as pharate adults, showing a larval-like epidermis and reduced proliferation of histoblast cells (Schleich et al., 2014) . Subsequent experiments using both RNAi in S2 cells and the knockout allele in larvae showed that DENR is required, along with its interacting partner MCT-1, for the proper expression regulation of a subset of transcripts required for cell cycle progression and growth. In particular, the loss of *DENR* reduces expression of the insulin receptor and makes larvae less sensitive to insulin signaling (Schleich et al., 2014) , thus implicating DENR in the regulation of the insulin signaling pathway.

***Synteny***

The target gene, *DENR ,* occurs on chromosome X in *D. melanogaster* and is flanked upstream by *CG13002* and *CG4880* and downstream by *RNA polymerase III subunit I* ( *Polr3I* ) and *Nitrogen permease regulator-like 2* ( *Nprl2* ). The *tblastn* search of *D. melanogaster* DENR-PB (query) against the *D. yakuba* (GenBank Accession: GCA_000005975.1 ) Genome Assembly (database) placed the putative ortholog of *DENR* within scaffold chromosome X (CM000162.2) at locus LOC6525601 ( XP_002101436.1 )— with an E-value of 3e-51 and 59.66% identity. Furthermore, the putative ortholog is flanked upstream by LOC6525603 ( XP_002101438.1 ) and LOC6525602 ( XP_043062942.1 ), which correspond to *CG13002* and *CG4880* in *D. melanogaster* (E-value: 2e-95 and 1e-150; identity: 67.53% and 71.18%, respectively, as determined by *blastp* ; Figure 1A, A ltschul et al., 1990). The putative ortholog of *DENR* is flanked downstream by LOC6525600 ( XP_002101435.1 ) and LOC6525599 ( XP_002101434.1 ), which correspond to *Polr3I* and *Nprl2* in *D. melanogaster* (E-value: 7e-94 and 0.0; identity: 85.54% and 99.73%, respectively, as determined by *blastp* ). The putative ortholog assignment for *DENR* in *D. yakuba* is supported by the following evidence: The genes surrounding the *DENR* ortholog are orthologous to the genes at the same locus in *D. melanogaster* and local synteny is completely conserved, supported by results generated from *blastp* , so we conclude that LOC6525601 is the correct ortholog of *DENR* in *D. yakuba* ( Figure 1A ).

***Protein Model***

Based on the annotation of two mRNA isoforms with identical protein-coding sequences in *D. melanogaster* , *DENR* in *D. yakuba* is predicted to have one unique protein-coding isoform ( *DENR-PB* and *DENR-PA* ; Figure 1B ). mRNA isoforms ( *DENR-RB* ; *DENR-RA* ) contain three CDS each. Relative to the ortholog in *D. melanogaster* , the RNA CDS number and protein isoform count are conserved. The sequence of DENR-PB in *D. yakuba* has 98.41% identity (E-value: 7e-97) with the protein-coding isoform DENR-PB in *D. melanogaster* , as determined by *blastp* ( Figure 1C ). Box X in purple highlights a gap in the dot plot, indicating a lack of sequence similarity in that region ( Figure 1C ). Coordinates of this curated gene model of DENR-PA and DENR-PB are stored by NCBI at GenBank/BankIt (accession BK064477 and BK064478 , respectively **)** . These data are also archived in the CaltechDATA repository (see “Extended Data” section below).

## Methods

Detailed methods including algorithms, database versions, and citations for the complete annotation process can be found in Rele et al. (2023). Briefly, students use the GEP instance of the UCSC Genome Browser v.435 ( https://gander.wustl.edu ; Kent WJ et al., 2002; Navarro Gonzalez et al., 2021) to examine the genomic neighborhood of their reference IIS gene in the *D. melanogaster* genome assembly (Aug. 2014; BDGP Release 6 + ISO1 MT/dm6). Students then retrieve the protein sequence for the *D. melanogaster* target gene for a given isoform and run it using *tblastn* against their target *Drosophila* species genome assembly ( *Drosophila yakuba* ( GCA_000005975.1 )- Graveley et al., 2010)) on the NCBI BLAST server ( https://blast.ncbi.nlm.nih.gov/Blast.cgi , Altschul et al., 1990) to identify potential orthologs. To validate the potential ortholog, students compare the local genomic neighborhood of their potential ortholog with the genomic neighborhood of their reference gene in *D. melanogaster* . This local synteny analysis includes at minimum the two upstream and downstream genes relative to their putative ortholog. They also explore other sets of genomic evidence using multiple alignment tracks in the Genome Browser, including BLAT alignments of RefSeq Genes, Spaln alignment of D. melanogaster proteins, multiple gene prediction tracks (e.g., GeMoMa, Geneid, Augustus), and modENCODE RNA-Seq from the target species. Genomic structure information (e.g., CDSs, CDS number and boundaries, number of isoforms) for the *D. melanogaster* reference gene is retrieved through the Gene Record Finder ( https://gander.wustl.edu/~wilson/dmelgenerecord/index.html ; Rele et al *.,* 2023). Approximate splice sites within the target gene are determined using *tblastn* using the CDSs from the *D. melanogaste* r reference gene. Coordinates of CDSs are then refined by examining aligned modENCODE RNA-Seq data, and by applying paradigms of molecular biology such as identifying canonical splice site sequences and ensuring the maintenance of an open reading frame across hypothesized splice sites. Students then confirm the biological validity of their target gene model using the Gene Model Checker ( https://gander.wustl.edu/~wilson/dmelgenerecord/index.html ; Rele et al., 2023), which compares the structure and translated sequence from their hypothesized target gene model against the *D. melanogaster* reference gene model. At least two independent models for this gene were generated by students under mentorship of their faculty course instructors. These models were then reconciled by a third independent researcher mentored by the project leaders to produce the final model presented here. Note: comparison of 5' and 3' UTR sequence information is not included in this GEP CURE protocol.

## Extended Data

Description: A GFF, FASTA, and PEP of the model. Resource Type: Model. DOI: 10.22002/bg0b6-1z133
